# Pleiotropic Effects of Modified Citrus Pectin

**DOI:** 10.3390/nu11112619

**Published:** 2019-11-01

**Authors:** Isaac Eliaz, Avraham Raz

**Affiliations:** 1 Amitabha Medical Clinic and Healing Center, 398 Tesconi Ct, Santa Rosa, CA 95401, USA; 2 Departments of Oncology and Pathology, School of Medicine, Wayne State University and Barbara Ann Karmanos Cancer Institute, 4100 John R St, Detroit, MI 48201, USA; raza@karmanos.org

**Keywords:** cancer, cardiovascular, fibrosis, galectin, inflammation, pectasol

## Abstract

Modified citrus pectin (MCP) has a low-molecular-weight degree of esterification to allow absorption from the small intestinal epithelium into the circulation. MCP produces pleiotropic effects, including but not limited to its antagonism of galectin-3, which have shown benefit in preclinical and clinical models. Regarding cancer, MCP modulates several rate-limiting steps of the metastatic cascade. MCP can also affect cancer cell resistance to chemotherapy. Regarding fibrotic diseases, MCP modulates many of the steps involved in the pathogenesis of aortic stenosis. MCP also reduces fibrosis to the kidney, liver, and adipose tissue. Other benefits of MCP include detoxification and improved immune function. This review summarizes the pleiotropic effects of MCP.

## 1. Introduction

**Figure 1:**
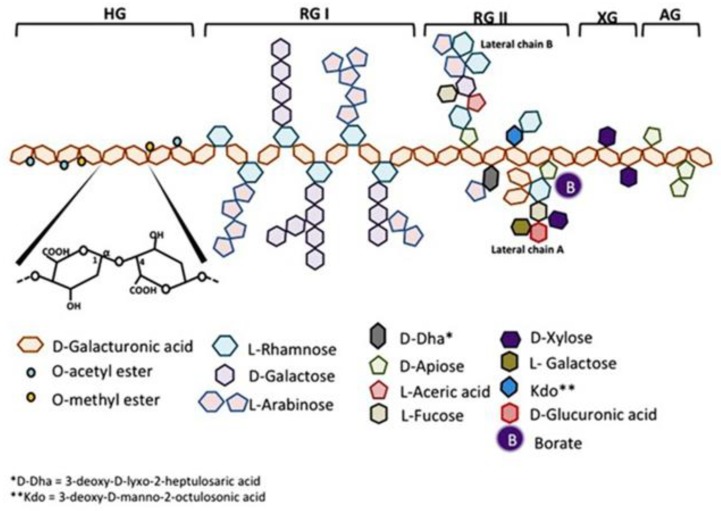
Schematic representation of pectin structure. AG, arabinogalactan; HG, homogalacturonan; RG, rhamnogalacturonan; XG, xylogalacturonan. It is reproduced with permission under a Creative Commons Attribution License (CC BY 4.0) https://creativecommons.org/licenses/by/4.0/ from Leclere, L.; Cutsem, PV.; Michiels, C.; Anti-cancer activities of pH- or heat-modified pectin. *Front Pharmacol*. 2013 Oct 8; 4:128 [1].

Citrus pectin is a soluble dietary fiber derived from the white pith of citrus fruit peels. Pectin is a large and complex molecule in its natural form, weighing 60–300 kilodalton (kDa), and containing a variable degree of (as much as ~70%) esterification. Pectins are a family of covalently linked galacturonic acid-rich polymers, with three identified central pectic polysaccharides regions: homogalacturonan (HG), rhamnogalacturonan-I (RG-I), and substituted galacturonans (GS). Among the GS is rhamnogalacturonan-II (RG-II), which is distinct from RG-I. RG-II has four types of structurally different oligosaccharides chains composed of 12 kinds of glycosyl residues [1] (Figure 1). Native pectin is not degraded during human digestion, and its large size prevents intestinal absorption [2,3]. However, when citrus pectin is modified (MCP) with a specific pH and heat-controlled enzymatic treatment to yield a product with a low molecular weight of <15 kilodaltons (kDa) and a degree of esterification under 5%, it can be absorbed from the small intestinal epithelium into the circulation [3]. The health benefits of MCP are increasingly recognized and summarized in this review and Table 1.

## 2. Galectin-3

**Figure 2:**
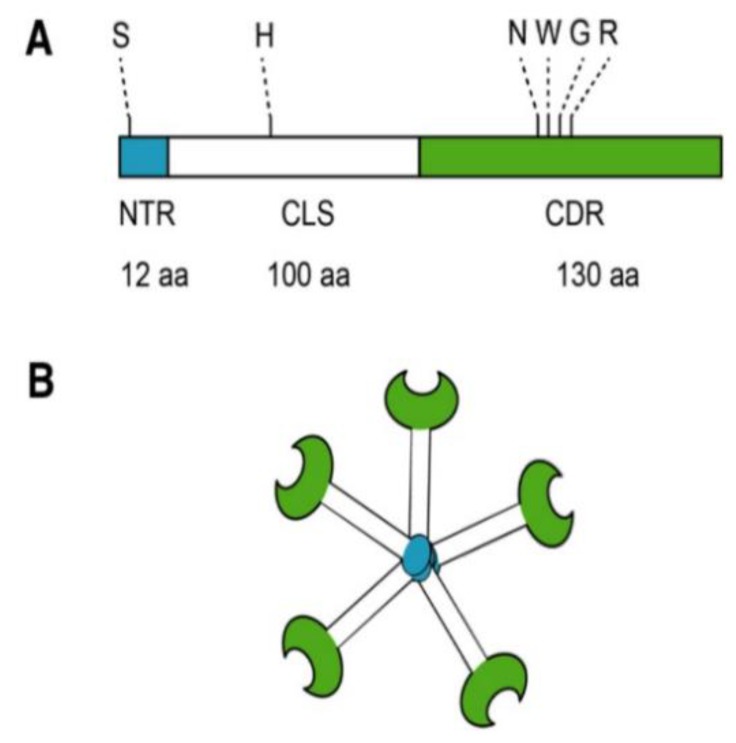
Structure of Gal-3. (**A**) Gal-3 protein structure consists of an N terminal Domain (NTD), which has an N terminal region of 12 amino acids (aa) and contains a serine 6 (S) phosphorylation site. The carbohydrate recognition domain (CRD) of 130 aa comprises the C-terminal and contains the anti-death motif or Asp-Trp-Gly-Arg (NWGR); (**B**) Pentameric structure of Gal-3. Reproduced with permission under a Creative Commons Attribution License (CC BY 4.0) https://creativecommons.org/licenses/by/4.0/. Clementy, N.; Piver, E.; Bisson, A.; André, Clémentine, A.; Bernard, A.; Pierre, B.; Fauchier, L.; Babuty, D. (2018). Galectin-3 in Atrial Fibrillation: Mechanisms and Therapeutic Implications. Int J Mol Sci. 2018 Apr; 19(4): 976 [49].

Most biomedical reports of MCP focus specifically on its antagonism of galectin-3 (Gal-3). Located in the nucleus, cytoplasm, outer cell surface, and extracellular space, Gal-3 is a member of a β-galactoside-binding lectin family [49]. Galectin-3 is a unique chimeric galectin containing a single carbohydrate recognition domain (CRD) of 130 amino acids at the COOH terminal with a unique 12 amino acid NH2-terminal domain (NTD). The CRD also contains what has been referred to as the anti-death motif or Asp-Trp-Gly-Arg (NWGR) due to sequence similarity to anti-apoptotic B-cell lymphoma-2 (Bcl-2) protein [50]. In the NTD, the serine 6 can be phosphorylated by casein kinases 1 and 2, contributing to nuclear translocation and a reduction of affinity to its ligands. Connecting the CRD and the NTD is a collagen-like sequence (CLS) domain consisting of about 100 amino acids and contains a Pro-Gly-Ala-Tyr-rich repeat motif. This long tail allows for unique pentameric oligomerization and contains the collagenase cleavable H-domain (Figure 2) [51]. Galectin-3 plays a prominent role in the progression of cancer and fibrotic diseases [52].

Regarding the former, the damaging effects of Gal-3 are due to its ability to accelerate the rate-limiting steps of metastasis [53]. Regarding the latter, the detrimental effects are due to its ability to bind matrix proteins such as cell surface receptors (integrins), collagen, elastin, and fibronectin and form cross-linking lattices in the extracellular matrix (ECM) [54]. Modified citrus pectin is abundant in β-galactose [53], which allows it to bind tightly to Gal-3 and modulate its bioactivity [55].

## 3. Cancer

Most of the morbidity and mortality associated with cancer is caused by metastasis, which is the migration of cancer from the site of primary tumor growth to distant organs and tissues. The metastatic cascade contains several rate-limiting steps that are modulated by Gal-3 and, in turn, by MCP as well [53]. The first step for neoplastic cells is to survive apoptosis that is associated with the loss of anchorage (anoikis) following escape from the primary tumor and intravasation. Galectin-3 protects cancer cells from anoikis [56,57] by causing a cell cycle arrest at the late G1 phase, which is an anoikis-insensitive point [56]. MCP has been shown to downregulate cyclin B and cdc2 in human prostatic JCA-1 cells [58], which may cause an accumulation of cancer cells in G2/M, thereby inducing apoptosis.

The next rate-limiting step in metastasis involves tumor cell arrest in distant organ microvasculature. Galectin-3 has been shown to mediate metastatic cell adhesion to the endothelium [59,60,61,62,63]. MCP was demonstrated to inhibit tumor cell adhesion to the endothelium as well as cancer cell homotypic aggregation involved in metastatic cell arrest in distant organs and the formation of intravascular metastatic deposits [59,64,65,66,67,68].

The third rate-limiting step in metastasis involves a forking point where tumor cells can either proliferate inside organ microvessels until the metastatic tumor outgrows the blood vessel and invades distant organ parenchyma [69], or extravasate before starting secondary tumor growth. Invasive propensity involves a series of tumor cell interactions with ECM proteins associated with the basement membrane and target organ stroma. MCP has been shown to reduce Gal-3-mediated tumor cell interactions with ECM proteins such as laminin [66]. Also, citrus pectin polysaccharides dose-dependently decreased the invasion through matrigel of human endothelial cells [67], of MDA-MB-231 human metastatic breast carcinoma cells [70], and human buccal metastatic cells [70].

After the initial parking in distant organs and extravasation, the overwhelming majority of cancer cells undergo apoptosis caused by various factors, and only ~2% survive and lead to micrometastasis [71]. Clonogenicity survival of early metastatic colonies is, therefore, the fourth rate-limiting step in metastasis. Galectin-3 protects cancer cells from different types of apoptosis by acting on mitochondrial pathways [72,73,74]. Some have suggested that MCP could undo Gal-3 anti-apoptotic function, thereby reducing the clonogenicity survival of cancer cells [74]. MCP dose-dependently inhibited the clonogenicity survival of hemangiosarcoma cells, and this was associated with increased tumor cell apoptosis [75].

Micrometastasis that transforms into clinically relevant secondary tumors eventually comes to depend on the development of new blood vessels via angiogenesis, the fifth and final rate-limiting step in metastasis. Galectin-3 promotes angiogenesis by serving as a chemoattractant for endothelial cells and inducing endothelial cell motility, invasion through matrigel, and capillary tube formation [67,76]. MCP was found to thwart chemotaxis of human endothelial cells toward Gal-3 dose-dependently, and inhibit in vitro capillary tube formation by endothelial cells dose-dependently [76]. The administration of MCP also reduced angiogenesis and spontaneous metastasis in vivo in tumor-bearing mice [76].

MCP can also modulate cancer cell resistance to chemotherapy. Most anti-neoplastic drugs work by inducing tumor cell apoptosis via the mitochondrial apoptosis pathway. As mentioned above, Galectin-3 dampens this pathway [77,78,79,80,81]. It also directly affects the sensitivity of cancer cells to chemotherapeutic drugs such as cisplatin [79,81,82], staurosporine [79], etoposide [81], bortezomib [83], dexamethasone [83], and doxorubicin (Dox) [75]. MCP has been demonstrated to reduce Gal-3 anti-apoptotic function and thereby reverse multiple myeloma cell resistance to bortezomib and enhance the response to apoptosis induced by dexamethasone [83]. Also, MCP substantially increased sensitivity to Dox-induced apoptosis in hemangiosarcoma cells [75].

Beyond this, MCP has been shown to induce apoptosis in cancer cells by itself through a caspase-8-to caspase-3 signaling cascade in the absence of change to mitochondrial membrane potential [83].

The reports of the anti-cancer benefits with the administration of MCP have continued. MCP inhibited the growth and metastasis of implanted colon cancer in mouse spleen [84]. It also induced cytotoxicity in both androgen-dependent and -independent prostate cancer cells in vitro [17]. Furthermore, MCP synergizes with Dox in the treatment of prostate carcinoma DU-145 and LNCaP cells by decreasing the viability and proliferation of cells [16]. When combined with paclitaxel (PTX), MCP increased caspase-3 activity and the percentage of human SKOV-3 ovarian cancer cells in subG1 [15]. Also, MCP inhibited the invasive potential of highly metastatic human breast (MDA-MB-231) or prostate (PC-3) cancer cells, when combined with a breast or prostate cellular health supplement, respectively [14]. A MCP-alginate probiotic dramatically inhibited precancerous lesions [13]. MCP reversed epithelial-mesenchymal transition, reduced cell proliferation, and increased suppression of anti-apoptotic proteins (Bcl-xL and survivin), with the collective action of promoting caspases-mediated apoptosis and inhibiting tumor cell growth [12]. MCP also inhibited tumor growth via the induction of cell cycle arrest and apoptosis in urinary bladder cancer cells in vitro and in vivo [11].

Moreover, MCP reduced prostate cancer cell viability and synergistically enhanced cell sensitivity to ionizing radiation [10]. Inhibition with the MCP of extracellular Gal-3 decreased colon cancer cell migration [9]. Also, the MCP abrogation of Gal-3 attenuated kidney tissue apoptosis and protected against the progression of renal fibrosis in cisplatin-induced nephrotoxicity [8]. Synergy with MCP and PTX to kill human ovarian cancer cell line (SKOV3) multicellular tumor spheroid through abrogation of signal transducer and activator of transcription 3 activity reduced expression of its downstream target HIF-1α, reduced integrin mRNA levels, and subsequently decreased AKT activity [7].

The size and domain structures of the MCP affect its anti-cancer properties. Using a more random heat (autoclaving) modification method to produce MCP, the enrichment of de-esterified homogalacturonan oligomers and the type one arabinogalactans (AG-I) and rhamnogalacturonan (RG-I) depletions in MCP smaller than 3 kDa, or the increase in AGI and decrease in RGI in MCP between 10 and 30 kDa, promoted anti-cancer behaviors by inhibiting migration, aggregation, and proliferation of cancer cells [85]. MCP, with its known specification for a low degree of esterification, low molecular mass, and a high percentage of RG-II domains, lends itself to an effective adjuvant oncological and immune therapy.

Clinical trials with the use of MCP have shown positive results. An open-labeled Phase II pilot study evaluated patients with biopsy-confirmed adenocarcinoma of the prostate, which were untreated at baseline, and had low but gradually rising prostate-specific antigen (PSA) levels (<10 ng/mL). Patients took MCP at a dose of 18 capsules per day (14.4 g) for 12 months. PSA doubling time (PSADT) was extended in 70% of patients [6]. The results indicate a slower cancer progression and possibly even prolongation of life.

An initial pilot trial investigated seven patients who had either relapsed after or failed prior treatment for prostate cancer (PSA range 0.63 to 7.50). The daily dosage of MCP was 15 g. A positive response (more than 30% lengthening of PSADT) was found in 4/7 patients, one patient had a partial response, one patient had stable disease, and one patient did not respond. All patients survived a 3-year follow-up [86].

An open labeled clinical trial examined patients with various solid tumors in an advanced state of progression. Treatment cycles consisted of 15 g of MCP daily for eight weeks. Six patients out of 29 (20.7%) had an overall clinical benefit response (pain, functional performance, weight change), as well as an improvement of quality of life. Eleven out of 49 (22.5%) showed stable disease (SD) after two cycles, and six out of 49 patients (12.3%) had SD for a period longer than 24 weeks. One patient suffering from metastasized prostate carcinoma experienced a halving of serum PSA after 16 weeks of therapy, as well as increased clinical benefit and quality of life, and decreased pain [5].

In an additional study, patients with detected circulating tumor cells received integrative therapy, which included advice on diet and exercise, supplementing with MCP, and supplementing with other products, including curcumin, green tea, garlic extract, vitamin D, medicinal mushroom extract, black cumin seed, artemisinin, and other unnamed supplements. Circulating tumor cell count decreased as a result of this integrative therapy [4].

Finally, there are promising interim results of an open-labeled Phase II study evaluating patients with non-castrate non-metastatic biochemically relapsed prostate cancer, presented at the 2019 American Society of Clinical Oncology (ASCO)-Genitourinary (GU) Cancers Symposium. Thirty-four patients consumed MCP at 4.8 g × 3/day for six months. No patient had treatment-related grade 3/4 toxicity, while six patients had grade 1 side effects (gas and bloating). Of these patients, twenty-one (62%) had stabilization or decrease in PSA, and negative scans, 27 patients (79%) had stabilization or improvement in PSADT and no metastases on scans [87]. The study is continuing with those showing benefit at six months staying on treatment for an additional 12 months.

## 4. Fibrotic Diseases

### 4.1. Aortic Stenosis

Many of the steps involved in the pathogenesis of aortic stenosis (AS) are modulated by Gal-3 and, therefore, presumably also by MCP [88]. Galectin-3 transforms quiescent fibroblasts into myofibroblasts that produce and secrete matrix proteins such as collagen [89,90]. Galectin-3 also affects collagen maturation and cross-linking [91,92]. It also stimulates pro-inflammatory mediators [93]. In human cardiac fibroblasts, Gal-3 increases the production and secretion of interleukin (IL)-1β, IL-6, monocyte chemoattractant protein-1, collagen type I and type III, and fibronectin [31]. It also increases the activity of metalloproteinases-1, -2, and -9 [31]. Treatment with MCP abrogates these effects. In the vasculature, Gal-3 promotes arterial stiffness by enhancing the production and secretion of pro-fibrotic and pro-inflammatory markers in vascular smooth muscle cells [33]. Again, MCP reverses this effect. In endothelial cells, Gal-3 enhances the expression of inflammatory factors, chemokines, and adhesion molecules [94]. Galectin-3 also affects the cell surface expression and activation of vascular endothelial growth factor receptor 2 in human endothelial cells, which contributes to plasma membrane retention and promotes angiogenesis [95]. In interstitial valve cells from the aorta, Gal-3 enhances the secretion of inflammatory and fibrotic mediators and increases the expression of calcification mediators [29]. The administration of MCP prevents these effects. MCP also prevented an increase in cardiac Gal-3, and normalized histological and molecular alterations in short-term AS [27].

### 4.2. Additional Cardiovascular Effects

Galectin-3 inhibition with MCP prevented cardiac inflammation and fibrosis associated with an excess of aldosterone levels independently of blood pressure levels [96]. Galectin-3 antagonism with MCP and aldosterone opposition reversed isoproterenol-induced left ventricular systolic dysfunction, thereby preventing the development of myocardial fibrosis in this mice model with selective cardiac hyperaldosteronism [30]. Blockade by MCP in a pressure overload-induced model, which increases Gal-3, showed amelioration of media thickness, fibrosis, and inflammation in aortic valve calcification [28]. It also reduced the size of atherosclerotic lesion areas by inhibiting the adhesion of leukocytes to endothelial cells [26].

Furthermore, MCP inhibited Gal-3 and decreased experimental abdominal aortic aneurysm development [25]. MCP reduced cardiac lipotoxicity and ameliorated cardiac mitochondrial damage in an obesity model [24]. Also, MCP restored the levels of cardiac peroxiredoxin-4 as well as prohibitin-2 levels and improved oxidative status [23]. Neurological impairments were prevented with MCP on post-aneurysmal subarachnoid hemorrhage, suggesting pleiotropic neuroprotective action, such as anti-neuroinflammatory and anti-apoptotic effects, beyond protecting the blood-brain barrier through inhibiting Gal-3 [22]. Inhibition mediated by the MCP of Gal-3 in mice prevented the pro-fibrotic and pro-inflammatory effects of cardiotrophin-1 [21]. Furthermore, MCP and perindopril comparably improved ischemic heart failure by downregulating Gal-3 and reducing myocardial fibrosis [20]. The blockade of Gal-3 with MCP prevented cardiac fibrosis, inflammation, and functional alterations [19].

### 4.3. Kidney

In a model of experimental acute kidney injury, all folic acid-treated mice lost weight while their kidneys enlarged secondary to the renal insult; MCP significantly lessened these gross changes, but this was not associated with changes in Gal-3 expression [36]. Via its blocking action on Gal-3, MCP protected against aldosterone-induced cardiac and renal fibrosis and dysfunction [32]. The inhibition of Gal-3 normalized renal Gal-3 levels as well as functional, histological, and molecular alterations in an obese and AS model, preventing renal fibrosis, inflammation, and damage with MCP treatment [35]. Moreover, MCP attenuated early renal impairment in spontaneously hypertensive rats as indicated by reduced albuminuria, improved renal function, and decreased renal fibrosis, epithelial-mesenchymal transition, and inflammation [34].

### 4.4. Additional Fibrotic Diseases

In a model of diet-induced obesity, MCP prevented adipose tissue fibrosis, inflammation, and the increase in adipocyte differentiation markers despite not affecting body weight, adipose tissue weights, or adiposity [37]. Liver fibrosis was alleviated with MCP and aided in hepatic regeneration, which may be mediated by an antioxidant effect [38].

## 5. Detoxification

To date, MCP has been used in four clinical studies of detoxification. Treatment with MCP increased urinary excretion of lead, arsenic, and cadmium in healthy volunteers, without side effects or depletion of essential elements [42]. In a case study of five patients, there was an average of 74% reduction in lead or mercury without side effects with the use of MCP alone or with a MCP/alginates combination [41]. Treatment with MCP dramatically decreased the levels of lead in blood and increased the levels of lead in urine in children hospitalized with lead toxicity [40]. Fecal uranium excretion was promoted by MCP/alginate supplement without side effects in a family with low-level chronic exposure from their environment and diet [39].

## 6. Immune Function

There are many reports of various effects on immunity with MCP. There was significant activation of T-cytotoxic and natural killer (NK) cells in blood cultures by MCP, and the NK-cells demonstrated functionality against K562 leukemic cells in culture. The presence of a low degree of methyl esterification and flexible low-molecular-weight pectin polymer enriched in saturated and unsaturated oligogalacturonic acids appear to be the immunostimulatory carbohydrates in MCP [48]. An additive effect of MCP in combination with cefotaxime against all six methicillin-resistant *Staphylococcus aureus* (MRSA) strains has been shown [47]. Honokiol, a purified extract from magnolia bark used in traditional Asian medicine and MCP, has been shown to have synergistic antioxidant activity and anti-inflammatory effects [46]. There was an inhibition of toxin-producing *Escherichia coli* adhesion and reduced Shiga toxin cytotoxicity with MCP [45]. Furthermore, MCP co-administration with live probiotic *L. acidophilus* ATCC 4356 supplement helped maintain or improve the integrity and population of the intestinal microbiota [44]. Finally, MCP has an immunomodulatory effect on the levels of cytokine secretion in the spleen of mice, which may be regulated by IL-4 [43].

## 7. Other Galectin-3 Inhibitors

There are other laboratory MCPs prepared by just heat and pH treatment. Heat by autoclaving MCP induced cell death in HepG2 and A549 cells. The induced cell death was different from classical apoptosis because there was no DNA cleavage [97]. Also, the delivery of autoclaved MCP reduced plaque volume in apolipoprotein E-deficient mice [98]. Renal cell carcinoma cells cotreated with autoclaved pectin and arsenic trioxide demonstrated increased apoptosis [99]. Synergistic treatment with S-trans, transfarnesylthiosalicylic acid and pH modified citrus pectin inhibited anaplastic thyroid cells proliferation in vitro by inducing cell cycle arrest and increased apoptosis rate [100]. pH modified citrus pectin also reduced the growth of solid tumors in balb-c mice [57].

Several Gal-3 inhibitors are also in pharmaceutical development. An injectable MCP in pharmaceutical development now abandoned called GCS-100 induced apoptosis in acute myeloid leukemia cells [101]. It also removed cell-surface Gal-3 from CD45, thus rendering diffuse large B-cell lymphoma cells susceptible to chemotherapeutic agents [102]. GCS-100 also detached Gal-3 from tumor-infiltrating lymphocytes and improved the cytotoxicity and secretion of different cytokines [103]. Furthermore, GCS-100 induced the inhibition of proliferation, the accumulation of cells in sub-G_1_ and G_1_ phases, and apoptosis with the activation of both the caspase-8 and -9 pathways [104]. GCS-100 enhanced calpain activation, which reduced the proapoptotic effect of Gal-3 [105].

Another Gal-3 inhibitor in pharmaceutical development is an inhalable formulation called TD139, a thiodigalactoside derivative. This inhibitor abrogated the susceptibility to natural killer T-cell-dependent hepatitis [106]. Pretreatment of wild-type C57BL/6 mice with TD139 lowered liver injury and led to milder infiltration of interferon-gamma and interleukin (IL)-17 and -4-producing cluster of differentiation (CD)4(+) T cells, and an increase in the total number of IL-10-producing CD4(+) T cells and F4/80(+) CD206(+) activating macrophages, and prevented the apoptosis of liver-infiltrating mononuclear cells [107]. TD139 blocked transforming growth factor-β-induced β-catenin activation in vitro and in vivo and lowered the late-stage progression of lung fibrosis after treatment with bleomycin [108].

Other pectin carbohydrate-based galectin inhibitors in drug development, injectables GR-MD-02 (galactoarabino-rhamnogalacturonan) and GM-CT-01 (galactomannan) resulted in a lowering of fibrosis with the reduction in the portal and septal Gal-3 positive macrophages and reductions in portal pressure [109]. The treatment resulted in sharp improvement in liver histology, with a significant decrease in non-alcoholic steatohepatitis (NASH) activity and collagen deposition; GM-CT-01 had an intermediate effect between the vehicle and GR-MD-02 [110].

## 8. Possible New Areas for MCP Research

Galectin-3 plays several prominent roles where there is little to no MCP research to date. One such role is bone pathophysiology [111]. Examples include acting as a cell marker and pro-survival factor in chondrocytes [112], a Runx2 target gene in osteoblasts [113], a cell marker in osteocytes [112], a biomarker for the pro-osteogenic capacity of mesenchymal stem cells [114], and a mediator of cell matrix adhesion in osteoclasts [115].

Galectin-3 has a mixed role in intestinal inflammation. Galectin-3 expression in the small bowel epithelial cells from Crohn’s disease patients is imbalanced and often significantly reduced [116]. Soluble Gal-3 acts as an activator of lamina propria fibroblasts [117]. It is also a substrate for matrix metalloproteinase-7, which may lead to delayed wound healing in chronic intestinal diseases [118]. Acute dextran sodium sulfate-induced colitis was ameliorated by Gal-3 in a mouse model [119]. Similarly, Gal-3 inhibited colonic mucosa inflammation by inducing regulatory T cells [120]. On the other hand, Gal-3 promotes the activation of NOD-like receptor family, pyrin domain containing 3 inflammasome and the production of IL-1β in macrophages [121].

Although the research on the relationship between Gal-3 and diabetes mellitus is also mixed, the overall suggest is that it has a pro-diabetic role. Key findings include the following: the delivery of Gal-3 to mice causes insulin resistance and glucose intolerance, whereas the inhibition of Gal-3, through either genetic or pharmacologic loss of function, improves insulin sensitivity in obese mice. Also, in vitro treatment with Gal-3 enhances macrophage chemotaxis, reduces insulin-stimulated glucose uptake in myocytes and 3T3-L1 adipocytes, and impairs the insulin-mediated suppression of glucose output in primary mouse hepatocytes [122]. In addition, Gal-3 directly activates peroxisome proliferator-activated receptor-γ and leads to adipocyte differentiation in vitro and in vivo [123]. Finally, circulating Gal-3 is positively associated with diabetes prevalence and incidence [124].

## 9. Conclusions

Clinical studies and preclinical research on the use of MCP have noted wide-ranging benefits. Much of the interest of MCP relates to its antagonism of Gal-3. As the Gal-3 research continues to identify novel mechanisms of disease progression, undoubtedly new benefits will be discovered for MCP. The advantageous effects of MCP are not limited to Gal-3 antagonism; other pleiotropic effects have been researched. Large-scale clinical trials are justified for examining the impact of MCP on robust clinical endpoints.

## Figures and Tables

**Table 1:** Pleiotropic effects of modified citrus pectin.

Main Indication	Study Type	Disease Model	Species Studied	Reference	Summary of Results
Cancer	Clinical trial	Circulating tumor cells	Human	[4]	Nutrients with anti-carcinogenic properties could reduce circulating tumor cell count, and included curcumin, garlic, green tea, grape seed, MCP, and medicinal mushroom extract
Cancer	Clinical trial	Advanced solid tumors	Human	[5]	Clinical benefits and life quality with far advanced solid tumors
Cancer	Clinical trial	Prostate cancer	Human	[6]	PSADT extended in 70% of patients
Cancer	Preclinical	Ovarian cancer	In vitro	[7]	MCP enhanced the PTX effect on ovarian cancer cells MCTS through the inhibition of STAT3 activity
Cancer	Preclinical	Cisplatin-induced nephrotoxicity	Mouse	[8]	MCP-treated mice demonstrated decreased renal fibrosis and apoptosis
Cancer	Preclinical	Colon cancer	In vitro, in vivo, and ex vivo	[9]	MCP inhibition of extracellular Gal-3 decreases colon cancer cell migration
Cancer	Preclinical	Prostate cancer and radiation therapy	In vitro	[10]	MCP reduced prostate cancer cell viability and synergistically enhanced cell sensitivity to ionizing radiation
Cancer	Preclinical	Bladder cancer	In vitro and mouse	[11]	Remarkable inhibitory effects of MCP on urinary bladder cancer cell proliferation and survival in vitro and in vivo mainly through Gal-3
Cancer	Preclinical	Gastrointestinal cancer	Mouse	[12]	MCP effectively inhibits the growth and metastasis of gastrointestinal cancer cells, partly by down-regulating Bcl-xL and Cyclin B to promote apoptosis and suppress EMT
Cancer	Preclinical	Colonic carcinogenesis	Mouse	[13]	Modified *L. acidophilus* ATCC 4356 cell envelope improved the bioavailability and the anti-(colon) cancer effect of MCP
Cancer	Preclinical	Breast and prostate cancer	In vitro	[14]	Inhibits breast/prostate cancer cell migration and synergy with MCP
Cancer	Preclinical	Ovarian cancer	In vitro	[15]	MCP synergy with paclitaxel
Cancer	Preclinical	Prostate cancer	In vitro	[16]	MCP synergy with doxorubicin
Cancer	Preclinical	Prostate cancer	In vitro	[17]	MCP induced cell death and inhibition of the proliferation of prostate cancer
Cancer	Preclinical	Liver and colon cancer	Mouse	[18]	MCP inhibits liver metastasis of colon cancer
Cardiovascular	Preclinical	Myocardial infarction	Rat	[19]	MCP blockade of Gal-3 can prevent cardiac fibrosis, inflammation, and functional alterations
Cardiovascular	Preclinical	Ischemic heart failure	Rabbit	[20]	Perindopril and MCP comparably improve ischemic heart failure in rabbits by downregulating Gal-3 and reducing myocardial fibrosis
Cardiovascular	Preclinical	Myocardial fibrosis	Rat, mouse, and human	[21]	MCP -mediated Gal-3 inhibition in mice prevented the profibrotic and proinflammatory effects of cardiotrophin-1
Cardiovascular	Preclinical	Blood-brain barrier disruption	Mouse	[22]	MCP prevents post-Subarachnoid Hemorrhage blood-brain barrier disruption possibly by inhibiting Gal-3, of which the mechanisms may include binding to TLR4 and activating ERK1/2, STAT3, and MMP-9
Cardiovascular	Preclinical	Cardiovascular fibrosis	In vitro, in vivo, and ex vivo	[23]	The pharmacological inhibition of Gal-3 with MCP restored cardiac Prx-4 as well as prohibitin-2 levels and improved oxidative status in spontaneously hypertensive rats
Cardiovascular	Preclinical	Cardiac lipotoxicity	Rat	[24]	Gal-3 inhibition with MCP attenuates consequences of cardiac lipotoxicity induced by a high-fat diet, reducing total triglyceride and lysophosphatidylcholine levels
Cardiovascular	Preclinical	Abdominal aortic aneurysm	Mouse	[25]	Mice treated with MCP showed decreased aortic dilation, as well as elastin degradation, vascular smooth muscle cell loss, and macrophage content at day 14 post-elastase perfusion compared with control mice
Cardiovascular	Preclinical	Atherosclerotic lesions in apoE-deficiency	Mouse	[26]	MCP reduced the size of atherosclerotic lesions by inhibiting the adhesion of leukocytes to endothelial cells
Cardiovascular	Preclinical	Aortic stenosis	Rat	[27]	In short-term AS, the increase in myocardial Gal-3 expression associated with cardiac fibrosis and inflammation, alterations that were prevented by Gal-3 blockade with MCP
Cardiovascular	Preclinical	Cardiovascular fibrosis and aortic valve calcification	Rat	[28]	MCP treatment prevented the increase in Gal-3, media thickness, fibrosis, and inflammation in the aorta of pressure overload rats
Cardiovascular	Preclinical	Aortic stenosis	Human and ex vivo	[29]	Gal-3 expression was blocked in VICs undergoing osteoblastic differentiation using MCP
Cardiovascular	Preclinical	Cardiovascular LV fibrosis	Mouse	[30]	MCP reversed induced LV dysfunction of HF with cardiac hyperaldosteronism
Cardiovascular	Preclinical	Cardiac inflammation and fibrosis in experimental hyperaldosteronism and hypertension	Rat	[31]	MCP prevention of inflammation and fibrosis with hypertension
Cardiovascular	Preclinical	Heart fibrosis	Rat	[32]	MCP prevention of cardiac fibrosis
Cardiovascular	Preclinical	Vascular fibrosis	Rat	[33]	MCP reverses vascular hypertrophy and fibrosis
Kidney	Preclinical	Renal damage in spontaneous hypertension	Rat	[34]	The inflammatory mediators (monocyte chemoattractant protein-1, osteopontin, cd68, cd80, cd44, and cd45) were elevated in spontaneously hypertensive rats and attenuated by MCP
Kidney	Preclinical	Kidney fibrosis	Rat	[35]	In experimental models of mild kidney damage, the increase in renal Gal-3 expression paralleled with renal fibrosis and inflammation, while these alterations prevented with MCP
Kidney	Preclinical	Kidney fibrosis	Rat	[32]	MCP prevention of kidney fibrosis
Kidney	Preclinical	Acute kidney disease	In vitro	[36]	MCP inhibits renal fibrosis
Obesity	Preclinical	Adipose tissue remodeling	Rat	[37]	Despite no effect on body weight, adipose tissue weights or adiposity, MCP prevented adipose tissue fibrosis, inflammation and the increase in adipocyte differentiation markers in a model of diet-induced obesity
Obesity	Preclinical	Adipose tissue remodeling/fibrosis	Rat	[31]	MCP prevented an increase in pericellular collagen, adipose tissue inflammation and differentiation degree of the adipocytes
Liver	Preclinical	Liver fibrosis	Rat	[38]	MCP attenuates liver fibrosis through an antioxidant effect, the inhibition of Gal-3, and the induction of apoptosis
Detoxification	Clinical trial	Chronic low-level uranium exposure	Human	[39]	MCP, after a post-treatment period of 6 weeks, decreased in fecal excretion of uranium found in 5 of 6 participants
Detoxification	Clinical trial	Child lead toxicity	Human	[40]	Detoxification from lead toxicity in hospitalized children
Detoxification	Clinical trial	Lead and mercury toxicity	Human	[41]	MCP lowered body burden of lead and or mercury and chronic ailment improvements
Detoxification	Clinical trial	Toxic metals	Human	[42]	MCP detoxification of lead, cadmium, arsenic, and mercury
Immune	Preclinical	Immuno-modulation	Mouse	[43]	CP and mainly MCP have an immunomodulatory effect on the levels of cytokine secretion in the spleen of mice with a pro-inflammatory potential
Immune	Preclinical	Probiotic	Mouse	[44]	The number of fecal lactobacilli in the MCP alginate probiotic-treated mice significantly increased
Immune	Preclinical	Shiga toxin producing *E. Coli*	In vitro	[45]	MCP inhibits adhesion of shiga toxin, reduces shiga toxin cytotoxicity
Immune	Preclinical	Inflammation	In vitro	[46]	MCP: Honokiol (9:1) combination induced a synergistic effect on antioxidant activity suggesting that the mixture is significantly more efficient than individual compounds
Immune	Preclinical	*Staphylococcus aureus*	In vitro	[47]	MCP demonstrates in vitro antimicrobial activity alone and combination with cefotaxime against staphylococcus aureus.
Immune	Preclinical	Immune activation	Human blood and ex vivo	[48]	MCP significantly activated T-cells and natural killer cells

Abbreviations. apoE: Apolipoprotein E, AS: Aortic stenosis, CP: Citrus pectin, EMT: Epithelial-mesenchymal transition, ERK1/2: Extracellular signal-related kinase 1/2, Gal-3: Galectin-3, HF: Heart failure, HNK: Honokiol, LV: Left ventricular, MCP: PectaSol-C Modified citrus pectin, MCTS: multicellular tumor spheroid, MMP-9: Matrix metalloproteinase-9, Prx-4: Peroxiredoxin-4, PSADT: Prostate-specific antigen doubling time, PTX: Paclitaxel, STAT3: Signal transducer and activator of transcription 3, TLR4: Toll-like receptor 4, and VIC: Valvular interstitial cell.
